# Pain Perception, Brain Connectivity, and Neurochemistry in Healthy, Capsaicin-Sensitive Subjects

**DOI:** 10.1155/2020/9125913

**Published:** 2020-10-28

**Authors:** Stefanie Heba, Matthias Sczesny-Kaiser, Kirsten Sucker, Jürgen Bünger, Thomas Brüning, Martin Tegenthoff, Tobias Schmidt-Wilcke

**Affiliations:** ^1^Department of Neurology, BG University Hospital Bergmannsheil, Bochum, Germany; ^2^Institute for Prevention and Occupational Medicine of the German Social Accident Insurance (IPA), Institute of the Ruhr-University Bochum, Bochum, Germany; ^3^Department of Neurology, St. Mauritius Therapieklinik, Lehrkrankenhaus der Universität Düsseldorf, Germany; ^4^Institute of Clinical Neuroscience and Medical Psychology, University of Düsseldorf, Düsseldorf, Germany

## Abstract

Most of the occupational exposure limits (OELs) are based on local irritants. However, exposure to much lower concentrations of irritant substances can also lead to health complaints from workers. Exposure to irritants is often accompanied by strong unpleasant odors, and strong odors might have distracting effects and hence pose a safety risk. The findings obtained in human exposure studies with chemically sensitive, stressed, or anxious persons suggest that their ability to direct attention away from the odorous exposure and to focus on a cognitive task is reduced. In addition, after repeated odor exposure, these persons show signs of sensitization, i.e., difficulties in ignoring or getting used to the exposure. The question arises as to whether certain health conditions are accompanied by a change in sensitivity to odors and irritants, so that these persons are potentially more distracted by odors and irritants and therefore more challenged in working memory tasks than nonsusceptible persons. In our study, susceptible persons with sensory airway hyperreactivity (“capsaicin-sensitive”) respond more strongly to mechanical skin stimuli than controls and show altered network connectivity. Capsaicin-sensitive subjects have a lower pain threshold and thus are more sensitive to mechanical skin stimuli. The intrinsic functional connectivity of their saliency network is higher, and the lower the GABAergic tone of the thalamus, the higher their pain sensitivity to mechanical stimuli. It seems that the increased communication between resting-state networks promotes a stronger perception of the sensory input signal. The results can be used to inform about actual risks (i.e., attention diversion and increased risk of accidents) and “pseudo” risks such as odor perception without a negative impact on one's well-being. This way, uncertainties that still prevail in the health assessment of odorous and sensory irritating chemicals could be reduced.

## 1. Introduction

Capsaicin, a naturally occurring alkaloid in fruits of the genus Capsicum, may be used to study sensory mechanisms of pain processing: Capsaicin triggers a trigeminal reflex such as painful burning and stinging sensations in the eyes and upper respiratory tract. This chemoreception is mediated via receptors (i.e., the transient receptor potential channel vanilloid 1 (TRPV1)) expressed at the free uncapsuled endings of A-delta and C nerve fibers. These receptors can be activated by irritants such as chemicals, but also by changes in the temperature, pH, or endogenous inflammatory mediators as discussed by Brüning et al. [1]. According to Tran et al. [2], intradermally injected capsaicin induces neurogenic inflammation that reflects peripheral mechanisms, as well as secondary hyperalgesia which reflects sensitized central nociceptive neurons.

In research, patients suffering from sensory hyperreactivity (SHR) are diagnosed with the capsaicin provocation test [3, 4] in combination with a questionnaire, the Chemical Sensitivity Scale for Sensory Hyperreactivity (CSS-SHR, [5]). These “capsaicin-sensitive” subjects report negative responses and behavioral disruptions caused by odor-intensive irritants and show extensive coughing after inhaling capsaicin. A main feature of this syndrome is the absence of bronchial obstruction and bronchial hyperreactivity as measured by methacholine provocation [6], or IgE-mediated reactions [7, 8]. The prevalence of airway SHR in Sweden has been estimated to 6% in the adult population [6].

Possible mechanisms of capsaicin susceptibility comprise either the increase in receptor density or a lower threshold of activation [9]; furthermore, there is evidence for central sensitization [10] consistent with brain imaging studies of central pain processing [11].

About 40% of occupational exposure limits (OELs) in the USA are based on local irritant effects [12]. Yet, many chemicals also activate the sense of smell, but usually at much lower concentrations [13]. Therefore, the Occupational Safety and Health Administration (OSHA) regulated three substances (isopropyl ether, phenyl ether, vinyl toluene) based on their “obnoxious odor.” These limits were established based on health complaints from workers and the assumption that strong odors have distracting effects and therefore pose a safety risk [14]. As OELs are set to keep the average population from harm, the incorporation of additional uncertainty factors (UFs) is currently under discussion. To account for differences in susceptibility relating to age, sex, lifestyle, personality, or diseases, a UF of about “2” was suggested [15]. For the protection of subjects with asthma or other preexisting, common health problems, a UF of “10” was advised [6]. However, the use of an additional UF was considered unnecessary whenever OELs for sensory irritants are derived based on reliable data from controlled exposure studies with healthy volunteers [1].

The findings obtained in studies with SHR subjects and stressed or anxious persons suggest that their ability to direct attention away from the odorous exposure and to focus on a cognitive task is reduced. In addition, after repeated odor exposure, these persons show signs of sensitization, i.e., difficulties in ignoring the exposure or getting used to it [5, 16, 17].

That said, controlled human exposure studies are inconclusive about the distracting effects of malodorous sensory irritants on work performance. In a 4-hour whole-body exposure study with 1-octanol, male volunteers who described themselves as chemically sensitive performed worse in a divided attention task compared to control subjects [18]. An impairment of work performance was also found in an exposure study with propionic acid [19, 20], but not with 2-ethylhexanol [21], or cyclohexylamine [22]. Exposure studies with odorous but nonirritating concentrations, which are typical at indoor workplaces, suggest that it is in particular the subjective rating of the odor (e.g., intensity, unpleasantness, health threat) that interferes negatively with work performance (for a review see Nielsen and Wolkoff [15]).

Supporting evidence comes from a study by Juran et al. [23] where subjects performed a go/no-go flanker task under whole-body exposure to propionic acid, which found increased event-related potentials during erroneous responses in no-go trials. The authors concluded that the unpleasant odor increased cognitive demands, which were more related to stimulus processing than performance monitoring, and thus led to the impairment in response inhibition. In concordance, brain imaging studies suggest alterations in the central nervous system as underlying pathophysiological mechanisms, for example, greater reactions in regions relevant for pain and saliency detection [2, 24].

The disgusting odor of spoiled brewer's yeast was used to study the effect of negative emotions on cognition in healthy volunteers and resulted in decreased working memory performance as task complexity increased [25]. In a second study, only in half of the subjects the unpleasant odor had a deteriorating effect on working memory [26]. The affected subjects demonstrated greater activation in emotion-associated areas, whereas unaffected subjects showed greater activation in task-relevant areas and were able to effectively maintain or even increase this activation. The authors concluded that in susceptible individuals, the coping mechanisms to reduce the distracting effect of the unpleasant odor did not work, so these individuals were more concerned with their emotions than with the task.

One of the few studies examining network connectivity in the context of odor processing has shown that there is a close link between the olfactory network and the default mode network (DMN). During the processing of olfactory information, the DMN is deactivated, suggesting that olfactory perception consumes resources for processing, attention, and storage processes [27]. In another study, changes in connectivity between the saliency network (SAL) and the DMN were found in the context of increased susceptibility to distraction [28], comparable to changes in network connectivity which have been found, for example, in patients with chronic pain or anxiety. It is believed that people with an overactive SAL are more susceptible to distraction than others [29]. In a big picture, the question remains how interindividual differences in emotion/sensory processing interact with the cognitive performance and whether the underlying cerebral correlates reflect more state or trait characteristics. Our current study, before investigating the impact of distraction on task performance in SHR subjects, starts one step earlier and asks in how far SHR subjects are different. As stated above, distraction (either directly by external stimuli or indirectly by evoked emotions) is known to influence one's ability to stay focused. Also, some people are much more prone to or affected by distraction than others, and when distraction constantly exceeds personal cognitive capacity in occupational settings, it may result in a higher risk for accidents, chronic headache or similar stress-related symptoms, depression, burnout, etc.

In the present study, we utilized sensory hyperreactivity as a model of (maladaptive) sensory processing and aimed to investigate the differences between healthy control subjects without (CON) and those with capsaicin cough sensitivity (CAPS). The central question is whether persons who are sensitive to external stimuli leaving most people unharmed are different with respect to pain perception, brain connectivity, and neurochemistry in two central brain hubs. Considering the main presumptions about how CAPS reacts to external irritant substances, such as central sensitization, altered pain processing, and diminished resilience against external distractors, we reasoned these effects to be seen in at least two resting-state networks (RSN): the sensorimotor network (SMN) and the SAL. First, we chose the SMN as it conveys sensory and nociceptive input and, therein, opted for the thalamus being the central hub at a precortical stage as magnetic resonance spectroscopy (MRS) target. Second, we decided to address the SAL for further investigation, because of its involvement in increased susceptibility to distraction, as well as pain processing. Within the SAL, we chose the insula as MRS target due to its prominent role in interoception and pain perception. Third, to enable interpretation of potential SMN and SAL findings in the context of increased susceptibility and prior DMN-SMN findings in chronic pain patients, we decided to include the DMN for within- as well as between-network connectivity investigations.

We hypothesize CAPS to hold (i) stronger neuronal connectivity within the SAL and (ii) stronger connectivity between the SAL and other resting-state networks, such as the SMN. Additionally, we expect (iii) the DMN of CAPS to be less interconnected to the SAL and/or SMN. In comparison to CON, we assume CAPS to be more respondent to (iv) weaker sensory stimuli in general, as external stimuli are more salient due to the strongly connected SAL. On a neurochemical level, we assume (v) a lower degree of inhibition in both SMN and SAL of CAPS, which are reflected by lower concentrations of the inhibitory neurotransmitter gamma-aminobutyric acid (GABA) and/or higher concentrations of the excitatory neurotransmitter glutamate (GLU) in both thalamus and insula. We believe these incidents to be the underpinnings of increased cognitive demands which in turn lead to higher distractive effects of external sensory stimuli.

## 2. Methods

### 2.1. Subjects

In total, 21 subjects (all right-handed, 10 female) with no previous history of psychological disorders were enrolled in the study. The subjects gave their written informed consent and received monetary compensation at the end of the experiment. The experimental protocol was approved by the local ethics committee of the Ruhr-University Bochum (Reg.-No. 4897-14) and was performed in accordance with the Declaration of Helsinki. All subjects completed an initial medical screening including a questionnaire-based anamnesis, tests for urinal cotinine (nicotine consumption), a methacholine provocation test (bronchial hyperreactivity), a Sniffin' Sticks test (olfactory screening; Burghart Medizintechnik, Wedel, Germany, [30]), blood serum levels of immunoglobulin E (type I hypersensitivity), pulmonary plethysmography (lung capacity), and a 12-stage capsaicin inhalation test. Out of the 21 subjects tested, 7 subjects were sensitive to capsaicin at stage 10 or higher. On subject level, age- and gender-matched individuals not responsive to capsaicin were drawn from the remaining 14 subjects and served as control. This resulted in 14 subjects (10 females; aged 23.8 ± 3.5 years) included in the analyses.

### 2.2. Thermal and Mechanical Quantitative Sensory Testing

Quantitative sensory testing is a well-established and broadly used method in clinical neurology to gauge sensory (mis-)perception. It follows a fixed protocol and requires specially trained personnel. Here, we followed the guidelines of Rolke and colleagues [31] and refer to his publication for details on data acquisition and analysis. QSTs were performed exclusively by one experienced experimenter. All sensory measurements were obtained from the palmar left lower arm, proximal to the wrist crest. Heat pain (HPT), cold pain (CPT), mechanical pain thresholds (MPT), and mechanical pain sensitivity (MPS) were acquired according to the standard clinical QST protocol [31–35]. Warmth (WDT) and cold detection thresholds (CDT) were obtained as additional control measures, to ensure normal nonnociceptive somatosensory function. Within the QST framework, thermal thresholds are determined using a method of limits. To this end, increasing and decreasing temperatures were applied to the skin with a thermal stimulator (MSA, Somedic, Hörby, Sweden), and the participants were instructed to indicate the onset of HPT or CPT by button press. For all thermal thresholds, six, instead of three, stimulus repetitions were performed to reduce between-subject variance. MPTs were determined using a staircase method. Five increasing and five decreasing trains of pinprick (MRC Systems, Heidelberg, Germany) stimuli were applied to the skin in an alternating fashion, whereas the participant was instructed to categorize the stimuli as noxious or nonnoxious. Mechanical pain sensitivity (MPS) was assessed using pinprick forces of 8, 16, 32, 64, 128, 256, and 512 mN which were presented once per run in a pseudorandomized order. Each subject received five runs and was asked to rate each stimulus for pain sensitivity using a numerical rating scale with “0” indicating no pain and a rating of “100” indicating the worst pain imaginable. Reference data [35] was used to categorize pinpricks as suprathreshold (128, 256, 512 mN) or subthreshold (8, 16, 32, 64 mN). The average of pain ratings given to pinpricks in the suprathreshold (or “heavy”) and subthreshold (or “light”) categories was defined as MPS_heavy_ and MPS_light_, respectively. Temporal pain summation was tested with single 256 mN pinprick stimuli applied and rated, followed by a train of ten stimuli at 1 Hz applied to the same skin location and rated per train. This set of single and train stimuli was repeated five times in total at five different skin sites within the test region. The mean pain rating of trains divided by the mean pain rating to single stimuli was calculated as the wind-up ratio (WUR).

### 2.3. Magnetic Resonance Imaging and Spectroscopy Specifications

The participants were scanned on a Philips 3.0 T Achieva X scanner using a 32-channel head coil. High-resolution, T1-weighted, structural images (MPRAGE, TR/TE: 8.5/3.9 ms, flip angle: 8°, Field of View (FOV): 256 × 256 × 220 mm, voxel (vx)-size 1 mm^3^ isotropic) were acquired to enable anatomically guided MRS voxel placement and tissue segmentation.

The MEGA-PRESS [36] sequence was used to obtain GABA+-edited spectra from single-voxel acquisitions over the right thalamus (vx-size 30 × 30 × 25 mm^3^) and right anterior insular cortex (vx-size 45 × 25 × 20 mm^3^) with a TR/TE: 2000/68 ms, 14 ms sinc-Gaussian editing pulses applied at 7.46 ppm and 1.9 ppm, 320 acquisitions in total with 20 averages of OFFs and ONs scans interleaved every 16 scans, and spectral bandwidth of 2 kHz with a sampling rate of 2048 points. Regional saturation technique slabs were applied in order to suppress fat signals from the skull, whereas variable power radio frequency pulses with optimized relaxation delays were used for water suppression. A separate non-water-suppressed scan followed the acquisition. Macromolecules were not suppressed, and therefore, those at the 1.72 ppm resonance were also partially inverted by the 1.9 ppm editing pulse. Since this signal is coupled to the 3.0 ppm resonance [37], those macromolecules would also have been affected by the editing pulse and therefore contribute to the difference spectra. Thus, GABA+ in this study refers to GABA including macromolecules. MRS sessions were scheduled so as to avoid the effects of frequency drift on GABA+-edited MRS [38, 39].

GLU was measured with point-resolved spectroscopy (PRESS, TR/TE: 2000/30 ms, flip angle: 90°, 32 averages, spectral bandwidth of 2 kHz with a sampling rate of 2048 points) at voxel locations identical to the MEGA-PRESS acquisition.

For the acquisition of resting-state functional images (Gradient-echo EPI, TR/TE: 2500/35 ms, flip angle: 90°, FOV: 240 × 240 mm, 40 axial slices, slice thickness: 3 mm, 10% gap, 200 scans, five dummy scans, total acquisition time: 8 min 37 s), participants were instructed to remain immobile, close their eyes, and “not to think about anything in particular.”

### 2.4. Analysis of Magnetic Resonance Spectroscopy

The GABA+ concentration was calculated using the GABA analysis toolkit *Gannet* (version 2, [40]). The brain volumes within each thalamic and insular voxel (matching the MRS voxels) were segmented into the gray matter (GM), white matter (WM), and cerebrospinal fluid (CSF) fractions, using the segmentation routine implemented in the VBM8 toolbox (http://dbm.neuro.uni-jena.de/wordpress/vbm/download/, last accessed September 29, 2020) as part of SPM8. Institutional units for GABA+/H_2_O were corrected *post hoc* for voxel tissue fraction by calculating the ratio of GABA+ units and the sum of GM and WM fractions according to, and are stated as, CSF-corrected individual GABA+ values.

LCModel (v6.3-1) was used for PRESS spectra quantification (basis set: press_te30ms_3t_gsh_v3). The analysis was restricted to signals within the 0.2 to 4.2 ppm range. Yielding a low relative standard deviation of estimates, we decided to choose GLU over the glutamate/glutamine model estimation, despite the spectral overlap between these two molecules. The estimated GLU levels were referenced to creatine+phosphorcreatine.

### 2.5. Image Preprocessing

Preprocessing of resting-state functional images was performed with the preprocessing routine provided by the functional connectivity toolbox CONN (version 14.n; [41]), and included slice time correction, spatial realignment and unwarping, normalization to the SPM8 MNI template, interpolation to (2 mm)^3^ isotropic voxel, and smoothing with an isotropic 6 mm Gaussian kernel; the images were centered to mean. The acceptable limit for the head motion was 2 mm for translational movements and 0.5° for rotational movements.

### 2.6. Independent Component Analysis

For the independent component analysis (ICA) using the GIFT toolbox (version 3.0a; http://icatb.sourceforge.net/groupica.htm, last accessed February 5, 2020), no filtering and no denoising were applied during preprocessing. Data dimensionality was reduced by two principal component analysis (PCA) steps to 36 on the subject level and after concatenation of subjects and sessions to 25 which is the estimated number of components using the minimum description length (MDL) criteria. The InfoMax group ICA was performed to decompose the data into 25 independent components (IC). ICA was repeated 20 times using ICASSO [42], starting each time from a random initial point. The reliability of decomposition was validated by the ICASSO results showing compact clusters. Subject-specific spatial maps (SM) and time courses (TC) of independent components were reconstructed using the GICA3 back-reconstruction method. The reconstructed SM of single components was converted to *Z*-scores, thresholded based on the voxelwise *t*-statistics according to Allen and colleagues [43], and visually inspected to select the ICs of particular interest in this study: the sensorimotor (SMN), the saliency (SAL), and the default mode (DMN) network. Intensity normalization was subsequently done to improve the accuracy and test-retest reliability of the ICA output, thus converting data to percent signal change. The functional network connectivity (FNC) was calculated within the MANCOVAN Toolbox, as provided by GIFT. Further analysis included intracomponent functional connectivity (iFC) and intercomponent functional connectivity (FNC) as in Schlaffke and colleagues [44]. In short, iFC (within-network) was calculated as the subject-specific median value of the back-reconstructed maps within a given network. The higher the average component values, the stronger the iFC strength. The FNC (network-to-network) comparisons were despiked, detrended, and filtered, as per default in the GIFT Toolbox. The low-pass filter cutoff used was 0.1 Hz, consistent with the Nyquist frequency corresponding to a TR of 2.5 s. Individual IC time courses were log-transformed to obtain a normal distribution before statistical analyses. The average functional connectivity between the ICs of the three networks was extracted for each subject.

### 2.7. Statistics

All results are quoted as mean ± SEM unless stated otherwise. The data has been tested for normal distribution by means of the Kolmogorov-Smirnov tests including Lilliefors' correction for small data sets. Between-group comparisons of CAPS vs. control data were performed by means of paired *t*-tests as well as repeated measures analysis of variance (ANOVA; Statistics toolbox and in-house scripts; MATLAB, R2009a, The MathWorks, Inc., USA). All *p* values are reported uncorrected, unless stated otherwise. Therefore, Cohen's *d* is given in to help in interpreting the potential implications of the results. We used Cohen's *d* as a measure of effect size, with absolute values between 0 and 0.2 depicting small effect sizes, absolute values between 0.2 and 0.5 depicting medium effect sizes, and absolute values between 0.4 and 1.2 depicting large effect sizes [45]. Finally, we performed multiple linear regressions within the CON and CAPS groups separately, setting MPS_light_ as the outcome variable and network connectivity and neurochemistry as predictive variables.

## 3. Results

### 3.1. Quantitative Sensory Testing (QST)

The QST results are summarized in Table 1. Thermal detection and pain thresholds do not differ between the capsaicin-sensitive and control subjects. Mechanical pain thresholds are lower in CAPS compared to CON and go along with a higher pain sensitivity to light mechanical pinprick stimulation in CAPS. Pinprick forces exceeding 128 mN are rated equally by both experimental groups.

### 3.2. Neurochemistry

Local GABA+ and GLU concentration was measured in single 22.5 cm^3^ voxels of the right thalamus and the right insula cortex. Neither in the thalamus nor the insula cortex we observe a difference in GABA+ or GLU levels between groups (Table 2). A 2-by-2-factorial ANOVA on the GABA+ data with factor group (CAPS and CON) and voxel position (thalamus and insula) found no main effect of group (*F*(1, 1) = 1.46, *p* = 0.239) and no interaction with voxel position (*F*(1, 1) = 0.55, *p* = 0.467). The same holds true for GLU data, where no main effect (*F*(1, 1) = 0.93, *p* = 0.344) and no interaction (*F*(1, 1) = 0.04, *p* = 0.837) were found.

### 3.3. Intra-network and Network-to-Network Connectivity

**Figure 1:**
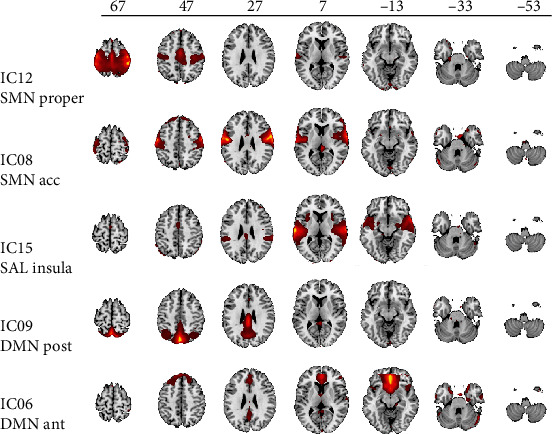
Spatial maps of independent components displayed on a T1 template. The top row gives the *z*-scale in mm MNI space. DMN ant: anterior part of default mode network; DMN post: posterior parts of default mode network; IC: independent component; SMN acc: accessory sensorimotor network; SMN proper: proper sensorimotor network; SAL insula: saliency network.

All resting-state networks of interest were represented by defined independent components (Figure 1). By visual inspection, IC 12 was defined as the proper SMN, comprising the pre- and postcentral gyrus (SMN_proper_); IC 8 as the accessory SMN comprising part of the bilateral secondary somatosensory cortex (SMN_acc_); IC 15 as the SAL including the anterior and posterior part of the insula cortex as well as part of the inferior parietal lobe; IC 9 as the posterior part of the DMN including the precuneus, posterior cingulate cortex, and the angular gyrus (DMN_post_); and IC 6 as the anterior part of the DMN including the anterior cingulate cortex and the middle frontal gyrus (DMN_ant_).

**Figure 2:**
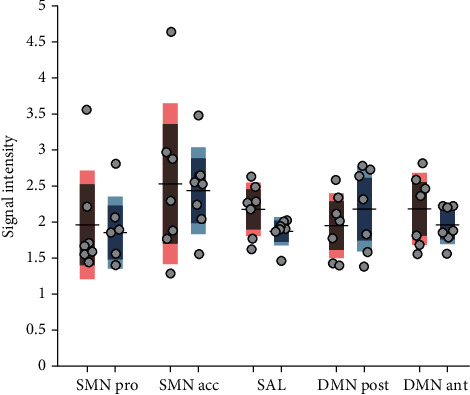
Median of signal intensities from back-reconstructed independent components, which is the physical measure of brain connectivity strength. The first column within each network gives the values of capsaicin-sensitive subjects (red), and the second column gives the values of the control subjects (blue). Horizontal lines indicate the mean, grey boxes indicate 1 standard deviation from the mean, and colored boxes give the 95% confidence interval. DMN ant: anterior part of default mode network; DMN post: posterior parts of the default mode network; SMN acc: accessory sensorimotor network; SMN pro: proper sensorimotor network; SAL: saliency network.

IFC was analyzed using a 5 (iFC) by 2 (group) multivariate general linear model ANOVA. We found no main effect of group (*F*(1, 1) = 1.591; *p* = 0.266, partial *η*^2^ = 0.499). To ensure not to miss any small effects due to sample size, we examined our *a priori* hypothesis and performed *post hoc* one-tailed *t*-tests on group differences in individual RSN. There were no significant differences in intrinsic functional connectivity strength in any of the networks after correcting for multiple tests (*p* > 0.3), yet interpreting the uncorrected *p* values together with their effect sizes gives hint towards a stronger SAL connectivity in CAPS compared to CON (*p* = 0.037, *p* > 0.1 after Bonferroni correction), whereas SMN proper and the accessory SMN do not differ between groups, as visualized in Figure 2. See Table 3 for detailed information on all analyzed components.

Individual network-to-network connections (Table 4) were analyzed using a 10 (FNC) by 2 (group) multivariate general linear model ANOVA. The main effect of the group reached significance (*F*(1, 1) = 16.318; *p* = 0.021, partial *η*^2^ = 0.982), indicating the FNC group differences. Between-subject effects revealed significant differences for the connectivity between DMN_ant_ and DMN_post_ (*F*(1, 12) = 7.255; *p* = 0.020, partial *η*^2^ = 0.377), such that CAPS show less connected DMN components than controls.

### 3.4. Multiple Linear Regression between Nociception, Neurochemistry, and Brain Connectivity

Four separate multiple linear regression models were conducted to see if neurochemistry and brain connectivity are capable to predict pain perception, i.e., MPS_light_.

*Model no. 1*. In the CON group, we found that intrinsic connectivity of the SAL in conjunction with GABA and glutamate level of the anterior insula explained a significant amount of the variance in mechanical pain sensitivity to light stimuli (*F*(3, 3) = 3.96, *F*_crit_ = 0.14, *R*^2^_adjusted_ = 0.59). Looking at single regressors, the analysis shows that only the iFC of the SAL predicted pain perception in the CON subjects significantly (beta = −0.54, *t*(6) = −3.37, *p* < 0.05), but the GABA (beta = −0.10, *t*(6) = −0.84, *p* = 0.46) or GLU level (beta = 0.32, *t*(6) = 0.93, *p* = 0.42) did not.

*Model no. 2*. The corresponding predictors in the CAPS group failed to explain any variance in the percept of mechanical pain (*F*(3, 3) = 0.36, *F*_crit_ = 0.79, *R*^2^_adjusted_ < 0.01).

*Model no. 3*. In the CON group, we found that the intrinsic connectivity of the SMN_proper_ together with the GABA and glutamate level of the thalamus did not account for variance in mechanical pain sensitivity to light stimuli (*F*(3, 3) = 0.66, *F*_crit_ = 0.63, *R*^2^_adjusted_ < 0.01).

*Model no. 4*. The corresponding predictors in the CAPS group explained a significant amount of the variance in mechanical pain sensitivity to light stimuli (*F*(3, 3) = 6.66, *F*_crit_ = 0.07, *R*^2^ adjusted = 0.74). With respect to the single regressors, the analysis shows that the thalamic GABA level did significantly predict pain perception in CAPS subjects (beta = −3.48, *t*(6) = −3.64, *p* < 0.05), but neither did iFC of the SMN_proper_ (beta = −0.08, *t*(6) = −0.38, *p* = 0.73), nor thalamic glutamate level (beta = 1.16, *t*(6) = 0.63, *p* = 0.58).

## 4. Discussion

The findings obtained in studies with SHR subjects, stressed or anxious persons, suggest that their ability to ignore external stimuli and keep focused on a cognitive task is reduced; this has also been reported for stimuli leaving most people unharmed or even unnoticed. In addition, after repeated exposure, these persons show signs of sensitization, i.e., difficulties in ignoring the exposure or getting used to it [5, 16, 17]. Therefore, deriving reliable exposure limits in occupational settings has become a challenge.

We utilized sensory hyperreactivity as a model of (maladaptive) sensory processing and investigated individuals with capsaicin cough sensitivity as a possibly susceptible subgroup of a healthy population. The central question is whether persons who are sensitive to external stimuli leaving most people unharmed are different with respect to pain perception, brain connectivity, and neurochemistry.

We could show that the capsaicin-sensitive subjects have a lower pain threshold and are more sensitive to light mechanical skin stimuli when compared to the control subjects. Further results from our study suggest that susceptible individuals respond more strongly to mechanical skin stimuli than controls due to altered network connectivity and neurochemistry in the somatomotor network, with thalamic GABA level being a significant predictor of MPS to light stimuli.

**Figure 3:**
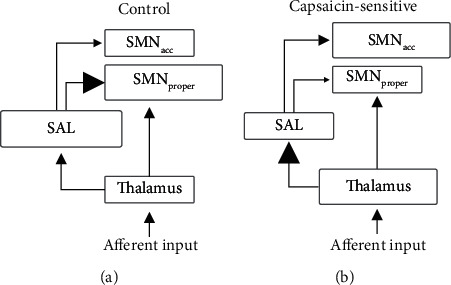
Visualization of the proposed changes in brain connectivity between the control (a) and capsaicin-sensitive subjects (b).

It appears that in the control group, an inappropriate overreaction to external stimuli is prevented by modulating the coupling between attention and sensory processing (Figure 3). In this context, there are studies stating that in humans, both primary (SI) and secondary (SII) somatosensory cortex are involved in pain processing. However, there are reports on a dichotomy between the sensory-discriminative perception of pain intensity, which is mainly processed in SI, and a more affective perception of pain intensity in SII [46]. Strikingly, we observe great overlap in the spatial maps of our network component SMN_proper_ with SI and of network component SMN_acc_ with SII.

One may assume that our observation of increased connectivity between the SAL and the SMN_proper_ in the control group promotes the interpretation of the afferent sensory input as harmless touch, as sensory information is mostly received and interpreted by SI. This association between the SAL and SMN_proper_ in CONs is reliable enough to predict the response to light mechanical stimuli. Our explanation is that the control subjects are concentrating more on the sensory-discriminative component of external stimuli without focusing on its unpleasant noxious element. In CAPS, however, both SMN_acc_ and SMN_proper_ are connected equally well to the SAL, so the preference for perceiving the stimuli as harmless is not granted (right panel in Figure 3).

Instead, a far more affective interpretation of incoming afferents by SII neurons might occur: data from nonhuman primate SII showed a complex firing pattern in response to threatening visual stimuli and their involvement in memorizing [47], detecting, and averting [48] noxious stimuli. In line with this, a stronger connection between the SAL and SMN_acc_ like we observe in the CAPS group should lead to pain sensitization, which is exactly what we see.

Referring to our association between the GABAergic tone of the thalamus and pain intensity in CAPS (i.e., the lower the thalamic GABA level, the more noxious a mechanical stimuli), it might be reasonable to assume low thalamic GABA level to result in an increased gain of afferent input on the subcortical level. In this case, with less inhibition in the thalamus, it would require less physical force by an external stimulus to trigger a cortical (i.e., cognitive) pain assessment. Supporting evidence for triggered (hyper-)sensitivity can be found in disease models such as migraine, asthma, and chronic regional pain syndrome (CRPS); for the SMN, it has been shown that diminished inhibition is associated with increased pain sensitivity [49]. Although speculative at this point, a disinhibition of the thalamus may result in nociception by switching from sensory-discriminative to SII-based affective stimulus perception.

More work is needed to disentangle the mechanisms between the SAL, SMN, and DMN, and how the neurochemical milieu in joint brain hubs (i.e., the thalamus) contributes to the observed behavioral differences. Testing a larger cohort in order to apply advanced analysis methods such as dynamic causal modeling would be the first step. Also, an interesting prospect for future studies would be to test patients with diseases associated with SHR such as migraine, fibromyalgia, or chronic back pain, with our multimodal study design.

## 5. Conclusion

In recent years, efforts were made to assess sensory irritants at workplaces, evaluate associated health complaints, and derive reliable exposure limits. There is evidence that individuals with respiratory diseases such as asthma or hay fever react stronger to volatile air pollutants than healthy subjects [50, 51]. With the study presented here, further insights can be gained on the evaluation of the adversity of irritants and odors. The results can be used to inform about actual risks (i.e., attention diversion and increased risk of accidents) and “pseudo” risks such as odor perception without a negative impact on one's well-being. This way, uncertainties that still prevail in the health assessment of odorous and sensory-irritating chemicals could be reduced.

## Figures and Tables

**Table 1:** Average QST parameters. Asterisks indicate log-transformed pain ratings prior to statistics.

QST parameter	All (*n* = 14)	CAPS (*n* = 7)	CON (*n* = 7)	*p* value	Cohen's *d*
CDT	30.2 (0.3)	29.9 (0.4)	30.5 (0.4)	0.359	0.51
WDT	34.5 (0.2)	34.7 (0.3)	34.3 (0.2)	0.278	-0.61
CPT	16.2 (2.0)	15.7 (3.6)	16.7 (2.1)	0.819	0.12
HPT	41.7 (1.0)	40.9 (1.7)	42.4 (1.3)	0.523	0.35
WUR^∗^	-0.743 (1.6)	-1.345 (3.0)	-0.141 (1.4)	0.719	0.20
MPT^∗^	1.409 (0.1)	1.294 (0.1)	1.524 (0.1)	0.030	1.32
MPS 8^∗^	-0.667 (0.2)	-0.335 (0.1)	-1.000 (0.4)	0.037	-1.26
MPS 16^∗^	-0.659 (0.2)	-0.340 (0.1)	-0.978 (0.4)	0.045	-1.19
MPS 32^∗^	-0.518 (0.1)	-0.173 (0.1)	-0.863 (0.3)	0.035	-1.27
MPS 64^∗^	-0.055 (0.02)	0.376 (0.1)	-0.485 (0.2)	0.006	-1.79
MPS 128^∗^	0.456 (0.1)	0.659 (0.3)	0.252 (0.1)	0.141	-0.84
MPS 256^∗^	0.635 (0.2)	0.804 (0.3)	0.466 (0.2)	0.206	-0.72
MPS 512^∗^	0.880 (0.2)	1.082 (0.4)	0.677 (0.3)	0.099	-0.96
MPS light^∗^	-0.475 (0.2)	-0.118 (0.3)	-0.831 (0.04)	0.014	-1.54
MPS heavy^∗^	0.657 (0.1)	0.848 (0.2)	0.465 (0.2)	0.137	-0.85

**Table 2:** Average GABA+ and GLU levels of the right thalamus and right insular cortex.

Neurochemistry	All (*n* = 14)	CAPS (*n* = 7)	CON (*n* = 7)	*p* value	Cohen's *d*
GABA+					
aINS	1.73 (0.08)	1.61 (0.12)	1.84 (0.09)	0.163	0.79
THAL	2.24 (0.08)	2.21 (0.07)	2.26 (0.16)	0.763	0.17
GLU					
aINS	1.40 (0.03)	1.43 (0.05)	1.38 (0.03)	0.463	-0.41
THAL	1.00 (0.03)	1.02 (0.03)	0.99 (0.04)	0.561	-0.32

**Table 3:** Single-subject intranetwork connectivity. DMN_ant_: anterior part of default mode network; DMN_post_: posterior parts of default mode network; SMN_acc_: accessory sensorimotor network; SMN_proper_: proper sensorimotor network, SAL: saliency network.

Subject ID	SMN_proper_	SMN_acc_	SAL	DMN_post_	DMN_ant_
CAPS1	1.44	4.64	2.49	2.01	2.46
CAPS2	1.59	1.77	1.77	1.40	1.55
CAPS3	3.56	2.97	2.28	1.77	2.36
CAPS4	2.21	2.88	2.27	2.11	2.59
CAPS5	1.71	1.88	2.18	2.58	2.82
CAPS6	1.55	2.29	2.63	2.34	1.81
CAPS7	1.67	1.29	1.62	1.43	1.68
CON1	2.81	3.48	1.91	1.58	2.22
CON2	1.89	2.53	2.01	2.64	1.85
CON3	1.40	1.55	1.46	2.78	1.88
CON4	1.85	2.65	2.02	1.83	2.20
CON5	1.56	2.04	1.87	1.38	1.79
CON6	1.40	2.55	1.94	2.32	2.22
CON7	2.06	2.24	1.90	2.73	1.56
CAPS	1.96 (0.28)	2.53 (0.42)	2.18 (0.14)	1.95 (0.17)	2.18 (0.19)
CON	1.85 (0.19)	2.43 (0.23)	1.87 (0.07)	2.18 (0.22)	1.96 (0.10)
*p* value	0.378	0.422	0.037	0.211	0.157
Cohen's *d*	-0.17	-0.11	-1.05	0.44	-0.56

**Table 4:** Network-to-network connections. CAPS: capsaicin-sensitive test group; CON: control group; DMN: default mode network; SAL: saliency network; SMN: sensorimotor network.

Subject ID	Age	SMN_prop_-SMN_acc_	SMN_prop_-SAL	SMN_prop_-DMN_post_	SMN_prop_-DMN_ant_	SMN_acc_-SAL	SMN_acc_-DMN_post_	SMN_acc_-DMN_ant_	SAL-DMN_post_	SAL-DMN_ant_	DMN_post_-DMN_ant_
CAPS1	27	0.40	0.36	0.02	0.21	0.47	-0.26	-0.02	-0.02	0.05	0.24
CAPS2	25	0.38	0.41	0.03	-0.013	0.54	-0.21	-0.05	0.04	0.27	0.29
CAPS3	27	0.37	0.04	-0.001	0.16	0.24	-0.37	-0.38	-0.19	0.05	0.10
CAPS4	30	0.78	0.35	-0.19	-0.22	0.53	-0.30	-0.25	0.09	-0.15	0.16
CAPS5	18	0.28	0.58	0.70	0.39	0.42	0.09	-0.05	0.43	0.24	0.11
CAPS6	29	0.77	0.37	0.03	0.29	0.29	-0.004	0.06	0.23	-0.11	-0.17
CAPS7	27	0.24	0.25	0.14	0.15	0.23	0.05	-0.13	0.05	0.16	0.07
CON1	27	0.93	0.33	-0.16	0.10	0.29	-0.33	-0.17	-0.06	0.13	0.26
CON2	23	0.31	0.26	0.21	-0.12	0.46	-0.84	-0.21	-0.18	-0.22	0.31
CON3	24	0.13	0.54	0.97	0.18	0.25	-0.24	0.10	0.19	0.22	0.29
CON4	28	0.53	0.58	0.21	0.25	0.40	-0.24	-0.10	-0.01	0.09	0.38
CON5	23	0.84	0.46	-0.10	0.36	0.23	-0.23	0.09	-0.27	0.25	0.30
CON6	29	0.31	0.48	0.67	0.29	0.29	-0.18	0.08	0.27	0.34	0.12
CON7	27	1.11	0.72	0.71	0.51	0.54	0.49	0.30	0.94	0.65	0.60
CAPS	26.1 (1.49)	0.46 (0.08)	0.34 (0.06)	0.10 (0.11)	0.14 (0.08)	0.39 (0.05)	-0.14 (0.07)	-0.12 (0.06)	0.09 (0.07)	0.07 (0.06)	0.12 (0.06)
CON	25.9 (0.94)	0.59 (0.14)	0.48 (0.06)	0.36 (0.16)	0.22 (0.08)	0.35 (0.04)	-0.22 (0.15)	0.01 (0.07)	0.12 (0.15)	0.21 (0.10)	0.32 (0.05)
*p* value	0.874	0.430	0.107	0.216	0.453	0.560	0.635	0.172	0.847	0.266	0.020
Cohen's *d*	-0.09	0.44	0.93	0.70	0.41	-0.32	-0.26	0.78	0.11	0.62	1.44

## Data Availability

Anonymized data can be accessed upon request to the corresponding author.

## References

[1] Brüning T., Bartsch R., Bolt H. M. (2014). Sensory irritation as a basis for setting occupational exposure limits. *Archives of Toxicology*.

[2] Tran M. T. D., Arendt-Nielsen L., Kupers R., Elberling J. (2013). Multiple chemical sensitivity: on the scent of central sensitization. *International Journal of Hygiene and Environmental Health*.

[3] Johansson A., Löwhagen O., Millqvist E., Bende M. (2002). Capsaicin inhalation test for identification of sensory hyperreactivity. *Respiratory Medicine*.

[4] Pullerits T., Ternesten-Hasséus E., Johansson E. L., Millqvist E. (2014). Capsaicin cough threshold test in diagnostics. *Respiratory Medicine*.

[5] Nordin S., Aldrin L., Claeson A. S., Andersson L. (2017). Effects of negative affectivity and odor valence on chemosensory and symptom perception and perceived ability to focus on a cognitive task. *Perception*.

[6] Johansson M. K. V., Johanson G., Öberg M. (2015). Evaluation of the experimental basis for assessment factors to protect individuals with asthma from health effects during short-term exposure to airborne chemicals. *Critical Reviews in Toxicology*.

[7] Fujimura M., Kasahara K., Yasui M. (1998). Atopy in cough sensitivity to capsaicin and bronchial responsiveness in young females. *The European Respiratory Journal*.

[8] Driessen A. K., McGovern A. E., Narula M. (2017). Central mechanisms of airway sensation and cough hypersensitivity. *Pulmonary Pharmacology & Therapeutics*.

[9] Midgren B., Hansson L., Karlsson J.-A., Simonsson B. G., Persson C. G. A. (1992). Capsaicin-induced cough in humans. *The American Review of Respiratory Disease*.

[10] Ando A., Smallwood D., McMahon M., Irving L., Mazzone S. B., Farrell M. J. (2016). Neural correlates of cough hypersensitivity in humans: evidence for central sensitisation and dysfunctional inhibitory control. *Thorax*.

[11] Andersson L. (2014). Multiple chemical sensitivity and persistent pain states are related, may be treated with similar procedures?. *Scandinavian Journal of Pain*.

[12] Dick R. B., Ahlers H. (1998). Chemicals in the workplace: incorporating human neurobehavioral testing into the regulatory process. *American Journal of Industrial Medicine*.

[13] van Thriel C., Triebig G., Bolt H. M. (2006). Editorial: evaluation of chemosensory effects due to occupational exposures. *International Archives of Occupational and Environmental Health*.

[14] Dalton P. H., Jaén C. (2010). Responses to odors in occupational environments. *Current Opinion in Allergy and Clinical Immunology*.

[15] Nielsen G. D., Wolkoff P. (2017). Evaluation of airborne sensory irritants for setting exposure limits or guidelines: a systematic approach. *Regulatory Toxicology and Pharmacology*.

[16] Andersson L., Bende M., Millqvist E., Nordin S. (2009). Attention bias and sensitization in chemical sensitivity. *Journal of Psychosomatic Research*.

[17] Andersson L., Claeson A.-S., Ledin L., Wisting F., Nordin S. (2013). The influence of health-risk perception and distress on reactions to low-level chemical exposure. *Frontiers in Psychology*.

[18] van Thriel C., Kiesswetter E., Blaszkewicz M., Golka K., Seeber A. (2003). Neurobehavioral effects during experimental exposure to 1-octanol and isopropanol. *Scandinavian Journal of Work, Environment & Health*.

[19] Hey K., Juran S. A., Schäper M. (2009). Neurobehavioral effects during exposures to propionic acid–an indicator of chemosensory distraction?. *Neurotoxicology*.

[20] Pacharra M., Schäper M., Kleinbeck S., Blaszkewicz M., Golka K., van Thriel C. (2016). Neurobehavioral effects of exposure to propionic acid revisited-does psychosocial stress interfere with distractive effects in volunteers?. *Neurotoxicology*.

[21] van Thriel C., Kiesswetter E., Schäper M. (2007). From neurotoxic to chemosensory effects: new insights on acute solvent neurotoxicity exemplified by acute effects of 2-ethylhexanol. *Neurotoxicology*.

[22] Juran S. A., van Thriel C., Kleinbeck S. (2012). Neurobehavioral performance in human volunteers during inhalation exposure to the unpleasant local irritant cyclohexylamine. *Neurotoxicology*.

[23] Juran S. A., van Thriel C., Kleinbeck S. (2013). Electrophysiological correlates of impaired response inhibition during inhalation of propionic acid. *Journal of Psychophysiology*.

[24] Andersson L., Claeson A.-S., Nyberg L., Nordin S. (2017). Short-term olfactory sensitization involves brain networks relevant for pain, and indicates chemical intolerance. *International Journal of Hygiene and Environmental Health*.

[25] Schneider F., Koch K., Reske M. (2006). Interaction of negative olfactory stimulation and working memory in schizophrenia patients: development and evaluation of a behavioral neuroimaging task. *Psychiatry Research*.

[26] Habel U., Koch K., Pauly K. (2007). The influence of olfactory-induced negative emotion on verbal working memory: individual differences in neurobehavioral findings. *Brain Research*.

[27] Karunanayaka P. R., Wilson D. A., Tobia M. J. (2017). Default mode network deactivation during odor-visual association. *Human Brain Mapping*.

[28] Götting F. N., Borchardt V., Demenescu L. R. (2017). Higher interference susceptibility in reaction time task is accompanied by weakened functional dissociation between salience and default mode network. *Neuroscience Letters*.

[29] Moran T. P., Moser J. S. (2015). The color of anxiety: neurobehavioral evidence for distraction by perceptually salient stimuli in anxiety. *Cognitive, Affective, & Behavioral Neuroscience*.

[30] Hummel T., Sekinger B., Wolf S. R., Pauli E., Kobal G. (1997). ‘Sniffin’ sticks’: olfactory performance assessed by the combined testing of odor identification, odor discrimination and olfactory threshold. *Chemical Senses*.

[31] Ziegler E. A., Magerl W., Meyer R. A., Treede R. D. (1999). Secondary hyperalgesia to punctate mechanical stimuli. *Brain*.

[32] Rolke R., Magerl W., Campbell K. A. (2006). Quantitative sensory testing: a comprehensive protocol for clinical trials. *European Journal of Pain*.

[33] Lang S., Klein T., Magerl W., Treede R.-D. (2007). Modality-specific sensory changes in humans after the induction of long-term potentiation (LTP) in cutaneous nociceptive pathways. *Pain*.

[34] Klein T., Stahn S., Magerl W., Treede R.-D. (2008). The role of heterosynaptic facilitation in long-term potentiation (LTP) of human pain sensation. *Pain*.

[35] Magerl W., Krumova E. K., Baron R., Tölle T., Treede R. D., Maier C. (2010). Reference data for quantitative sensory testing (QST): refined stratification for age and a novel method for statistical comparison of group data. *Pain*.

[36] Mescher M., Merkle H., Kirsch J., Garwood M., Gruetter R. (1998). Simultaneous in vivo spectral editing and water suppression. *NMR in Biomedicine*.

[37] Behar K. L., Boehm D. (1994). Measurement of GABA following GABA-transaminase inhibition by gabaculine: a 1H and 31P NMR spectroscopic study of rat brain in vivo. *Magnetic Resonance in Medicine*.

[38] Dydak U., Schär M. (2006). MR spectroscopy and spectroscopic imaging: comparing 3.0 T versus 1.5 T. *Neuroimaging Clinics of North America*.

[39] Harris A. D., Glaubitz B., Near J. (2014). Impact of frequency drift on gamma-aminobutyric acid-edited MR spectroscopy. *Magnetic Resonance in Medicine*.

[40] Edden R. A. E., Puts N. A. J., Harris A. D., Barker P. B., Evans C. J. (2014). Gannet: a batch-processing tool for the quantitative analysis of gamma-aminobutyric acid–edited MR spectroscopy spectra. *Journal of Magnetic Resonance Imaging*.

[41] Whitfield-Gabrieli S., Nieto-Castanon A. (2012). Conn: a functional connectivity toolbox for correlated and anticorrelated brain networks. *Brain Connectivity*.

[42] Himberg J., Hyvärinen A., Esposito F. (2004). Validating the independent components of neuroimaging time series via clustering and visualization. *Neuro Image*.

[43] Allen E. A., Erhardt E. B., Damaraju E. (2011). A baseline for the multivariate comparison of resting-state networks. *Frontiers in Systems Neuroscience*.

[44] Schlaffke L., Schweizer L. M., Rüther N. N. (2017). Dynamic changes of resting state connectivity related to the acquisition of a lexico-semantic skill. *Neuro Image*.

[45] Cohen J. (1988). *Statistical power analysis for the behavioral sciences*.

[46] Timmermann L., Ploner M., Haucke K., Schmitz F., Baltissen R., Schnitzler A. (2001). Differential coding of pain intensity in the human primary and secondary somatosensory cortex. *Journal of Neurophysiology*.

[47] Lenz F. A., Gracely R. H., Zirh A. T., Romanoski A. J., Dougherty P. M. (1997). The sensory-limbic model of pain memory. *Pain Forum*.

[48] Robinson C. J., Burton H. (1980). Somatic submodality distribution within the second somatosensory (SII), 7b, retroinsular, postauditory, and granular insular cortical areas of M. fascicularis. *The Journal of Comparative Neurology*.

[49] Lenz M., Höffken O., Stude P. (2011). Bilateral somatosensory cortex disinhibition in complex regional pain syndrome type I. *Neurology*.

[50] Jaén C., Dalton P. (2014). Asthma and odors: the role of risk perception in asthma exacerbation. *Journal of Psychosomatic Research*.

[51] Claeson A. S., Palmquist E., Lind N., Nordin S. (2016). Symptom-trigger factors other than allergens in asthma and allergy. *International Journal of Environmental Health Research*.

